# Hyperinflammation, T cells, and endotoxemia

**DOI:** 10.18632/oncotarget.5236

**Published:** 2015-08-22

**Authors:** Makoto Inoue, Mari L. Shinohara

**Affiliations:** Department of Immunology and Department of Molecular Genetics and Microbiology, Duke University School of Medicine, Durham, NC USA

**Keywords:** endotoxemia, hyperinflammation, antigen-independent, T cell migration, osteopontin

Endotoxemia and sepsis are systemic hyperinflammatory disorders triggered by microbial infection. When infections happen, host cells in the innate immune system quickly act to produce pro-inflammatory cytokines, such as tumor necrosis factor (TNF). Appropriate development of inflammatory responses is essential to protect hosts from microbial infections. However, when inflammatory responses overshoot, collateral organ damages occur in hosts. To avoid this, our immune system is equipped with a variety of mechanisms to downregulate immune responses. To control hyperinflammation in the innate immune system, adaptive immunity was not considered to be involved; because initiating adaptive immune responses takes time. However, others and we demonstrated that T cells, which are components of the adaptive immune system, inhibit hyperinflammation during endotoxemia and sepsis [1–4].

Inhibition of unleashed innate immune responses by T cells was first reported by Kim et al. [1]. The study showed that T cells suppress excessive production of TNFα and IFNγ during poly-I:C-induced endotoxemia [1]. Later on, Guarda et al. reported that T cells suppress IL-1β production by downregulating activities of NLRP1 and NLRP3 inflammasomes. We have recently reported detailed molecular and cellular mechanisms, by which T cells inhibit hyperinflammation during LPS endotoxemia [3, 4].

First critical finding for us was that adoptive transfer of total T cells from naïve mice successfully improved survival of *Rag2^−/−^* mice (mice lacking T and B cells) from endotoxemia by decreasing systemic TNF levels [3]. Importantly, transferred T cells did not need to be activated by antigens, suggesting that the T cells protected hosts in an antigen-independent fashion. Indeed, naïve T cells inhibited TNF production by LPS-treated macrophages without any cognate antigens in tissue culture; and the function was demonstrated in 24 hours, which is too early for T cell activation [3]. Importantly, direct contact between T cells and macrophages was essential for the T cell-mediated inhibitory function [3].

Next, we sought receptor(s) mediating the inhibitory mechanism. Because cognate antigens were not involved in the inhibitory mechanism by T cells, the involvement of MHC molecules was ruled out. Instead, interaction between CD40 on macrophages and CD40L on T cells turned out to be critical [3]. CD40 is constitutively expressed in macrophages. On the other hand, Tregs (Foxp3^+^CD4^+^) in naive mice and Foxp3^−^ CD4^+^ T cells in LPS-injected mice expressed CD40L; thus, CD4^+^ T cells do not need to be primed by antigens to express CD40L. Therefore, CD40 on macrophages can be stimulated by antigen-unexperienced T cells from mice going through LPS-endotoxemia [3]. In contrast to B cells, in which CD40 ligation alone stimulate NFκB activity, macrophages NFκB is neither activated nor inhibited by CD40 signaling alone [3].

Then, how CD40 signaling attenuates TNF production by macrophages stimulated with LPS? We found that, when macrophages are stimulated with LPS, additional CD40 signaling further enhanced IL-10 expression. IL-10 detected by macrophages in an autocrine fashion shortened the half-life of *Tnfa* mRNA. The key trigger was TLR4/CD40 dual stimulation [3]. Here is how enhancement of IL-10 expression is initiated: IRAK1 translocates into the nucleus, when CD40 is stimulated on top of TLR4 stimulation. Then, IRAK1 binds to the *Il10* promoter as a transcription factor [3]. Although IRAK1 is known to be a cytosolic molecule, previous reports had demonstrated that IRAK1 can translocate into nuclear when sumoylated [5, 6]. For IRAK sumoylation, TRAF2 and intracellular osteopontin (iOPN), which is as an adapter molecule in cytosolic signaling cascade [7], were required. Then, the role of TLR4 here is to provide activated IRF5 as a chaperon to sumoylated IRAK1 for translocation into the nucleus [3]. Therefore, with the TLR4/CD40 dual stimulation, IL-10 expression by macrophages is elevated. IL-10, now detected by IL-10 receptor on macrophages, destabilizes *Tnfa* mRNA; and eventually the levels of TNFα are downregulated.

Macrophages reside in different compartments from T cells in secondary lymphoid organs, such as spleen. How can macrophages possibly interact with T cells *in vivo*? We found that LPS “draws” T cells out from the T cell-rich regions to the red pulp, where macrophages are abundant, in the spleen at a very early stage of LPS endotoxemia [4]. We found that secreted OPN (sOPN) from macrophages recruits T cells around 3 hrs after LPS injection. Macrophages quickly produce OPN after LPS treatment, and attract T cells, which detect sOPN by integrin αv [4]. Indeed, neutralizing sOPN or blocking integrin αv antibody strongly inhibits T cell migration towards macrophages, increases serum TNF levels, and enhances susceptibility to endotoxemia [4]. We have mentioned the involvement of iOPN in the cytoplasm of macrophages in IRAK1 sumoylation. sOPN and iOPN are translational isoforms of OPN, and generally known to induce pro-inflammatory responses [7]. Yet, in endotoxemia, both sOPN and iOPN work as anti-inflammatory molecules [3, 4]. Thus, the role of the sOPN-integrin αv axis at a very early stage of endotoxemia is critical to recruit T cells towards macrophages and to initiate the T cell-macrophage contact for protecting hosts from TNF-mediated hyperinflammation via iOPN.

Even without antigen experience, T cells play a critical role in inhibiting hyperinflamamtion ongoing in the innate immune system. Our group and others reported a new inhibitory function of T cells in acute hyperinflammation. However, it might be possible that similar phenomena in controlling innate immunity by naive T cells occur in other pathological conditions.
